# Supplementary material for: Promoting nickel oxidation state transitions in single-layer NiFeB hydroxide nanosheets for efficient oxygen evolution
Source: Nat Commun. 2022 Oct 15;13:6094. doi: 10.1038/s41467-022-33846-0 (PMC9568589; doi:10.1038/s41467-022-33846-0)
Supplement: Supplementary file 1 — Supplementary Information [file 41467_2022_33846_MOESM1_ESM.pdf]

Supporting Information

Promoting Nickel Oxidation State Transitions in Single-Layer NiFeB Hydroxide Nanosheets for Efficient Oxygen Evolution

Yuke Bai,[1‡] Yu Wu,[2‡] Xichen Zhou,[1] Yifan Ye,[3] Kaiqi Nie,[4] Jiaou Wang,[4] Miao Xie,[2] Zhixue Zhang,[1] Zhaojun Liu,[1] Tao Cheng,[2]* and Chuanbo Gao[1]*

[1]*State Key Laboratory of Multiphase Flow in Power Engineering, Frontier Institute of Science and Technology, Xi'an Jiaotong University, Xi'an, Shaanxi 710054, China.*

[2]*Institute of Functional Nano & Soft Materials (FUNSOM), Jiangsu Key Laboratory for Carbon-Based Functional Materials & Devices, Joint International Research Laboratory of Carbon-Based Functional Materials and Devices, Soochow University, Suzhou, Jiangsu 215123, China.*

[3]*National Synchrotron Radiation Laboratory, University of Science and Technology of China, Hefei 230029, China.*

[4]*Institute of High Energy Physics, Chinese Academy of Sciences, Beijing 100049, China*

[‡]*These authors contributed equally to this work.*

**Corresponding authors: C.G. (email: gaochuanbo@mail.xjtu.edu.cn), T.C. (email: tcheng@suda.edu.cn)*

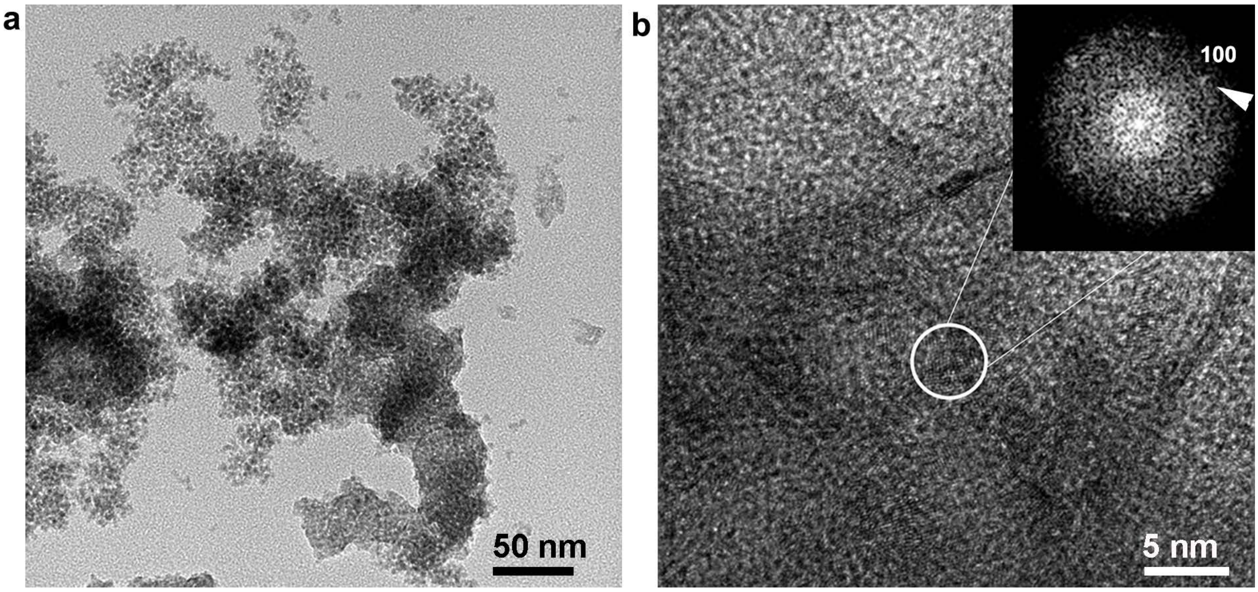

Figure S1. Morphological change during the transformation of NiFeB alloy nanoparticles into NiFeB hydroxide nanosheets by hydrolysis. (a) TEM image of the NiFeB alloy nanoparticles. (b) HRTEM image of the NiFeB hydroxide nanosheets. Inset: Fourier diffractogram, showing the (100) diffraction spot.

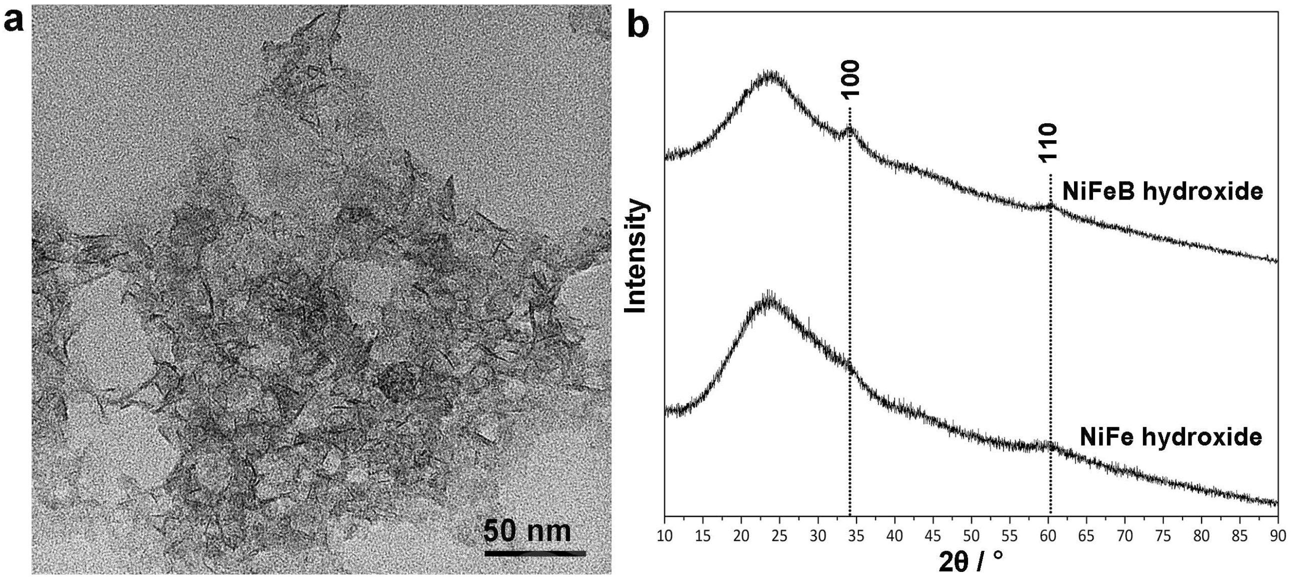

Figure S2. Characterization of the NiFe hydroxide nanosheets. (a) TEM image. (b) XRD patterns. XRD pattern of the NiFeB hydroxide nanosheets were presented for comparison. In-plane 100 and 110 diffractions are observable, suggesting a similar MO_6 structure in the single-layer nanosheets.

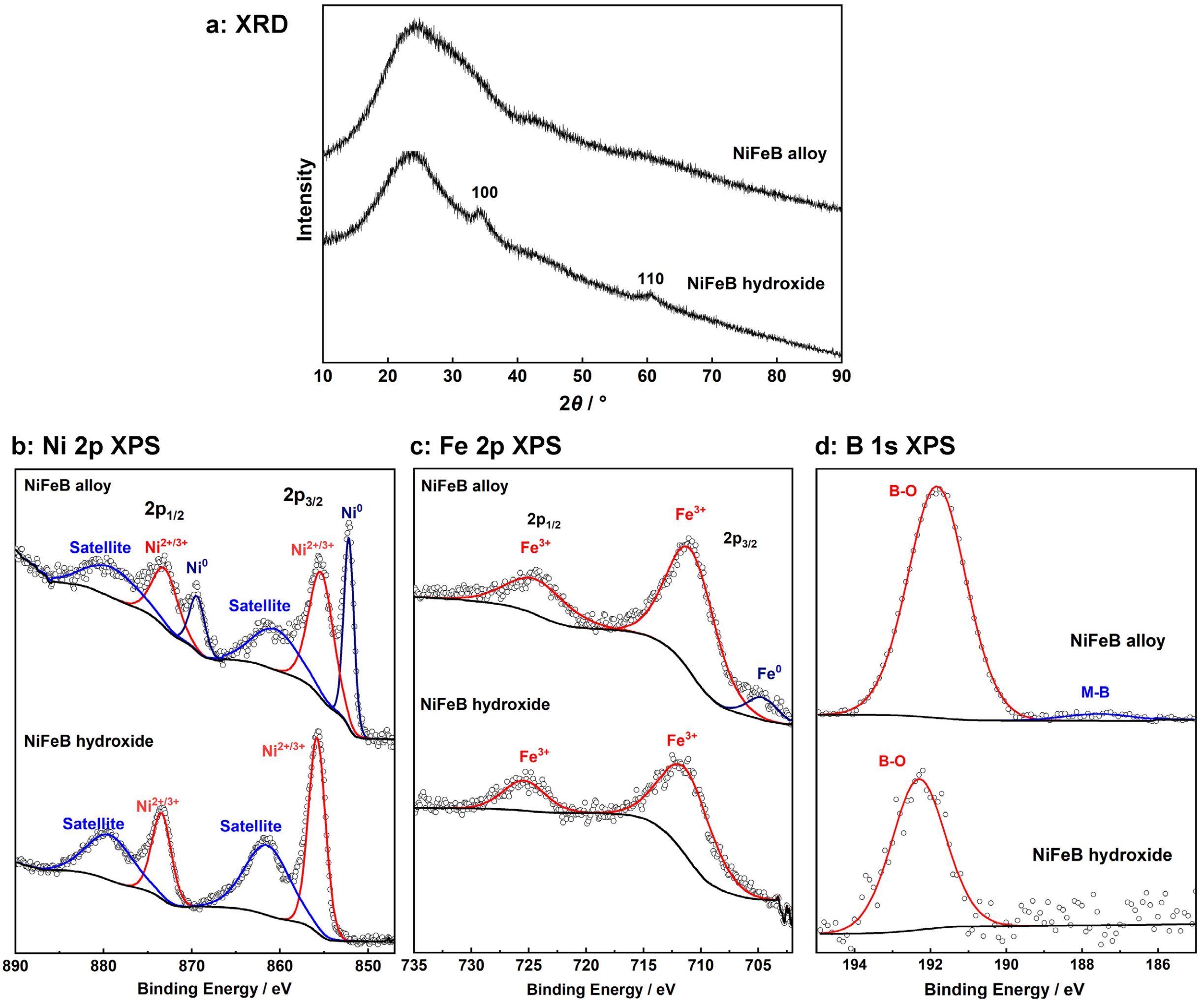

Figure S3. Conversion of the NiFeB alloy nanoparticles into NiFeB hydroxide nanosheets. (a) XRD patterns of the NiFeB alloy nanoparticles and the NiFeB hydroxide nanosheets. These results show that the amorphous NiFeB alloy nanoparticles were converted into a crystalline NiFeB hydroxide nanosheets by hydrolysis in KOH. (b–d) Ni 2*p*, Fe 2*p*, and B 1*s* XPS of the NiFeB alloy nanoparticles and the NiFeB hydroxide nanosheets. Zero-valent Ni^0, Fe^0, and B (M–B) exist in initially synthesized NiFeB alloy nanoparticles. After hydrolysis, the zero-valent species disappeared, showing only oxidized species, suggesting a complete conversion of the alloy nanoparticles into hydroxides.

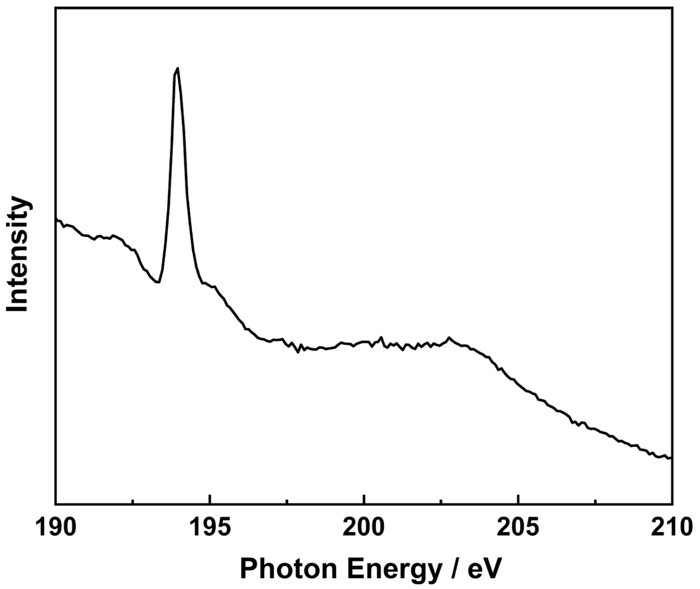

Figure S4. B *K*-edge XAS of the NiFeB hydroxide nanosheets. The sharp peak at 194 eV corresponds to the electron transition in a BO_3 group with a trigonal configuration.

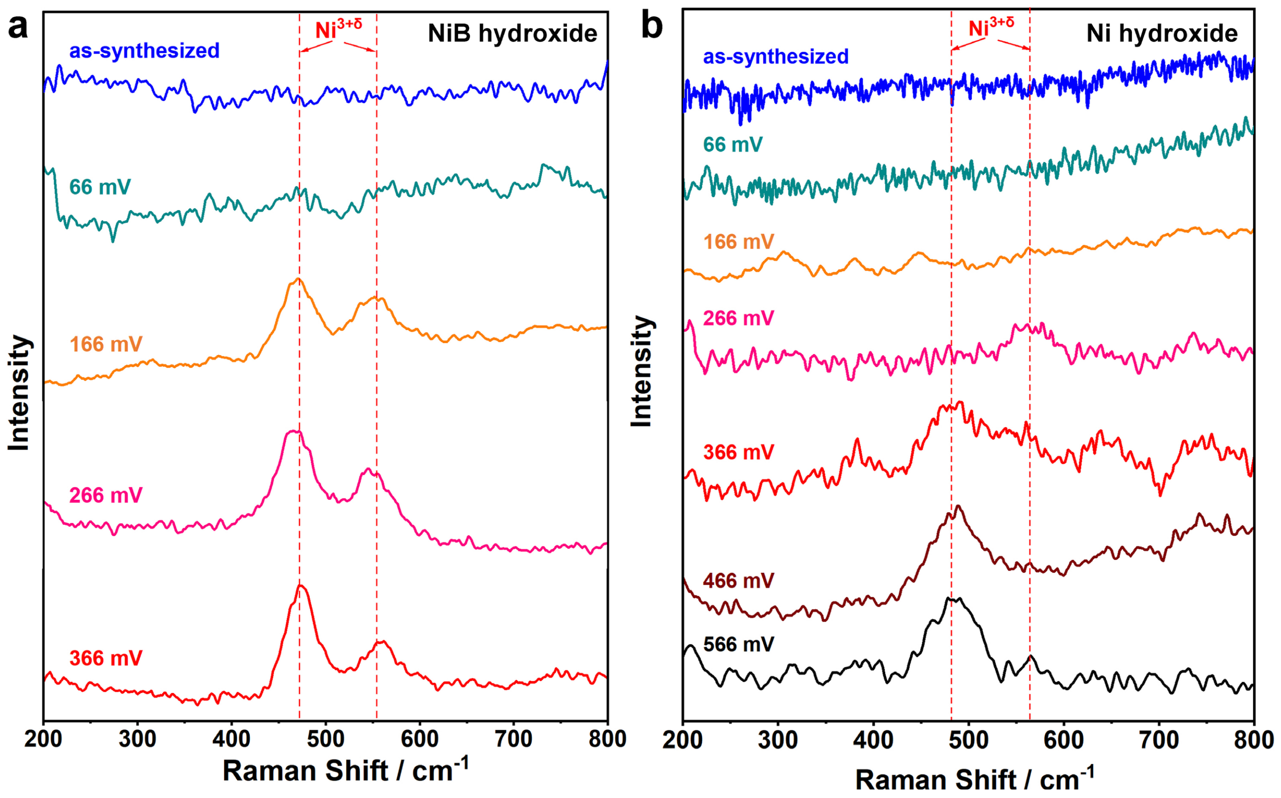

Figure S5. Raman spectra of the NiB hydroxide (a) and Ni hydroxide nanosheets (b) after they were maintained at different overpotentials in O_2-saturated KOH (1 M), showing the $Ni^{2+}(OH)_2 \rightarrow Ni^{3+\delta}OOH$ transition. Compared with the NiFeB hydroxide nanosheets (Figure 2a, oxidation state transition at 66 mV), the NiB hydroxide nanosheets require a higher overpotential to trigger the $Ni^{2+}(OH)_2 \rightarrow Ni^{3+\delta}OOH$ (166 mV), which suggests that Fe also contributes to the boosted oxidation of Ni in the hydroxide catalyst. By comparing the Raman spectra of the NiB and Ni hydroxide nanosheets, it is inferred that the incorporation of B into the Ni hydroxide nanosheets significantly promotes the $Ni^{2+}(OH)_2 \rightarrow Ni^{3+\delta}OOH$ transition, which is similar to the case observed in the NiFeB and NiFe hydroxide systems.

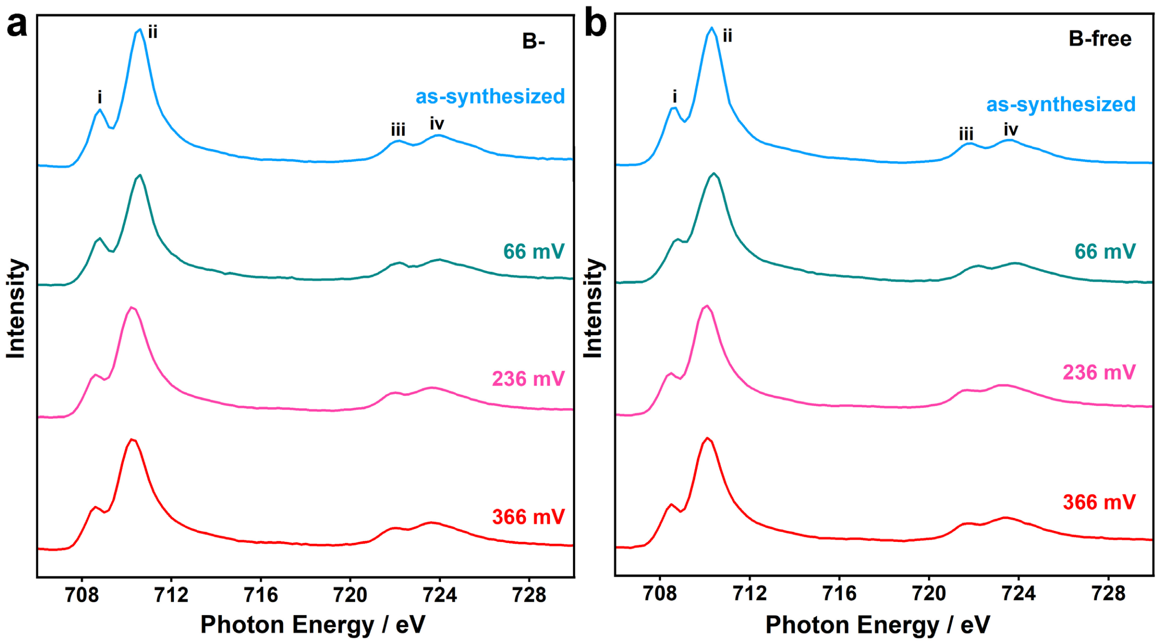

Figure S6. Fe $L_{3,2}$-edge XAS spectra of the NiFeB hydroxide (a) and B-free NiFe hydroxide nanosheets (b) after they were maintained at different overpotentials (66, 236, 366 eV). The XAS spectra of the as-synthesized samples are also presented for comparison. The peaks i and iii correspond to the Fe $2p \rightarrow 3d$ t_{2g} transition, and the peaks ii and iv correspond to the Fe $2p \rightarrow 3d$ e_g transition.

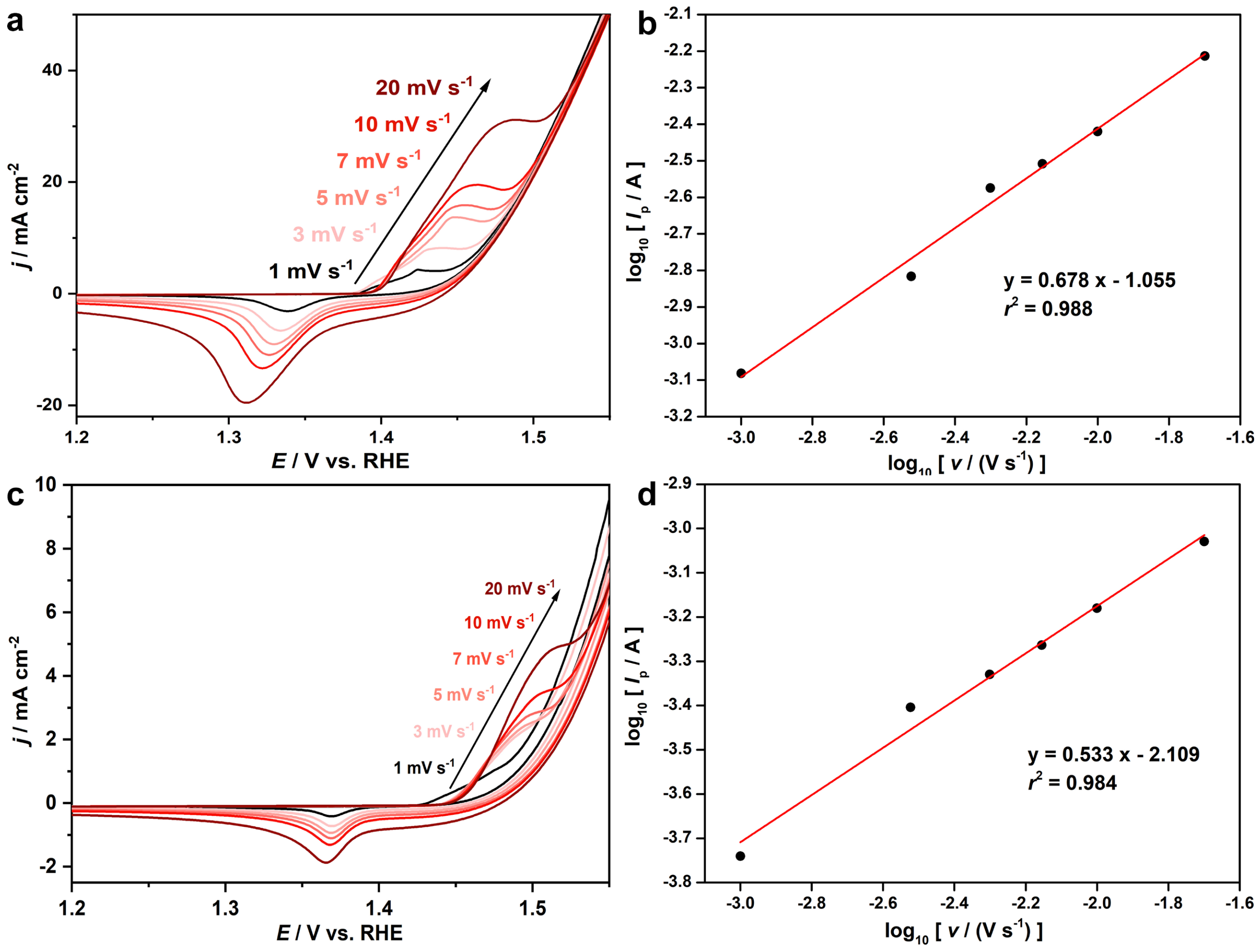

Figure S7. CV of the NiFeB (a, b) and NiFe hydroxide nanosheets (c, d) in O_2-saturated KOH (1 M) at different scan rates. (a, c) CV curves. (b, d) Relationship between the current density at the peak (~1.45 V) and the scan rate. The slopes were fitted to be ~0.5, suggesting that the peaks in the CV prior to the OER correspond to a Faraday process [$Ni^{2+}(OH)_2 \rightarrow Ni^{3+\delta}OOH$ transition] in the catalyst.

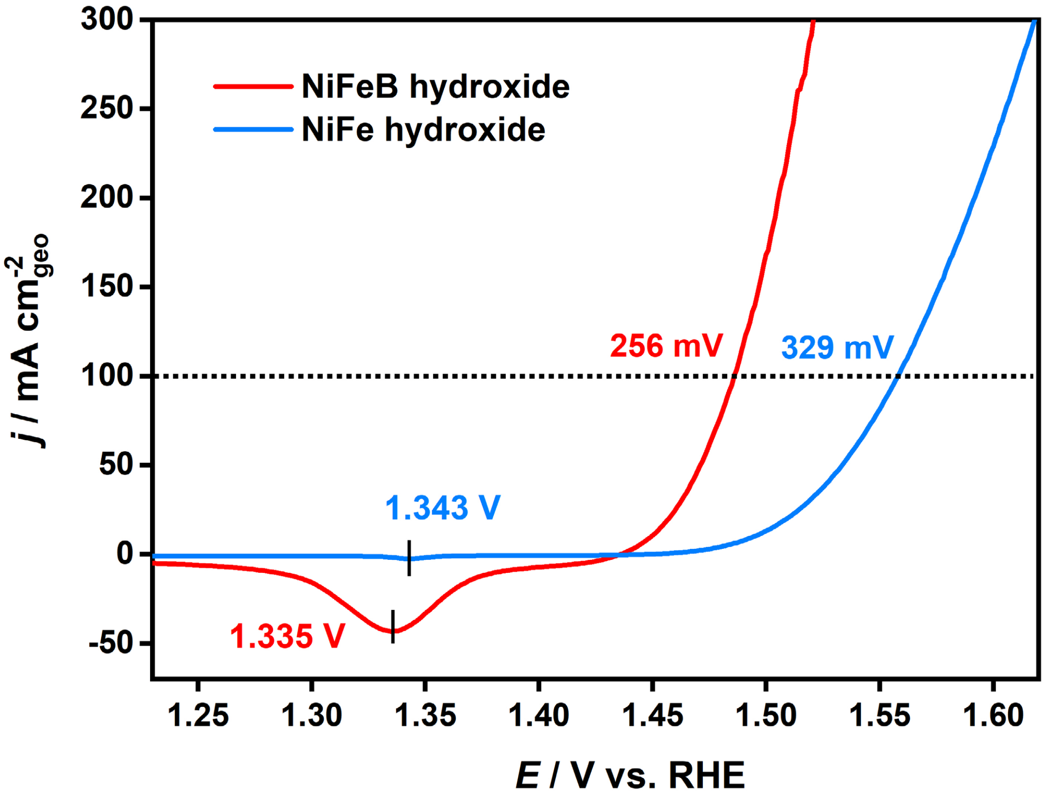

Figure S8. OER polarization curves of the NiFeB and NiFe hydroxide nanosheets obtained via a cathodic potential sweep (1.743–1.043 V vs RHE, 10 mV s^{-1}, 95% *iR* compensation). The current densities were normalized to the geometric area of the electrode (0.196 cm^2).

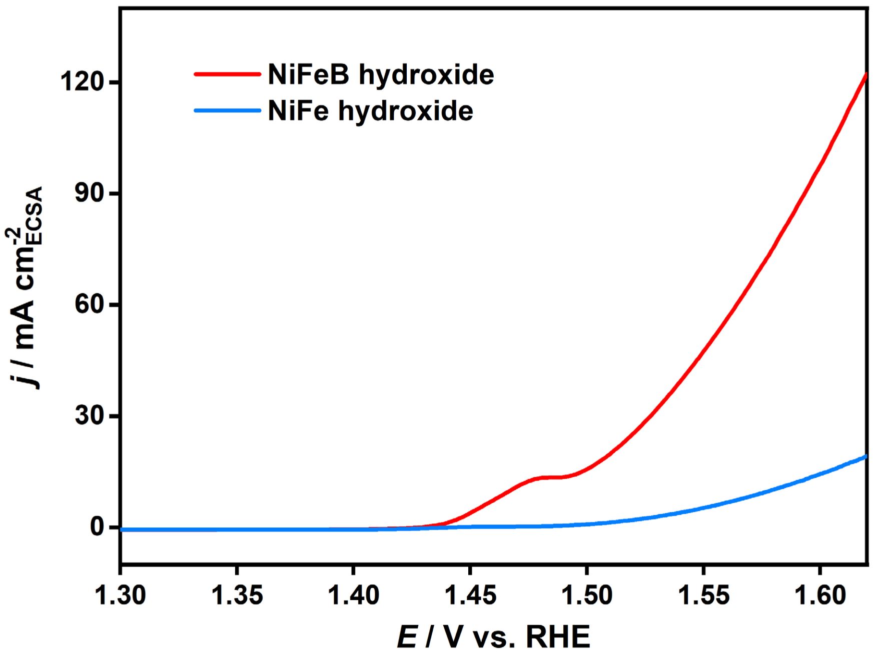

Figure S9. OER polarization curves of the NiFeB and NiFe hydroxide nanosheets without supporting carbon (95% *iR* compensation) with the current densities normalized to the ECSA. The ECSAs were calculated according to the double layer capacitance of the catalysts (details, see Figure S10).

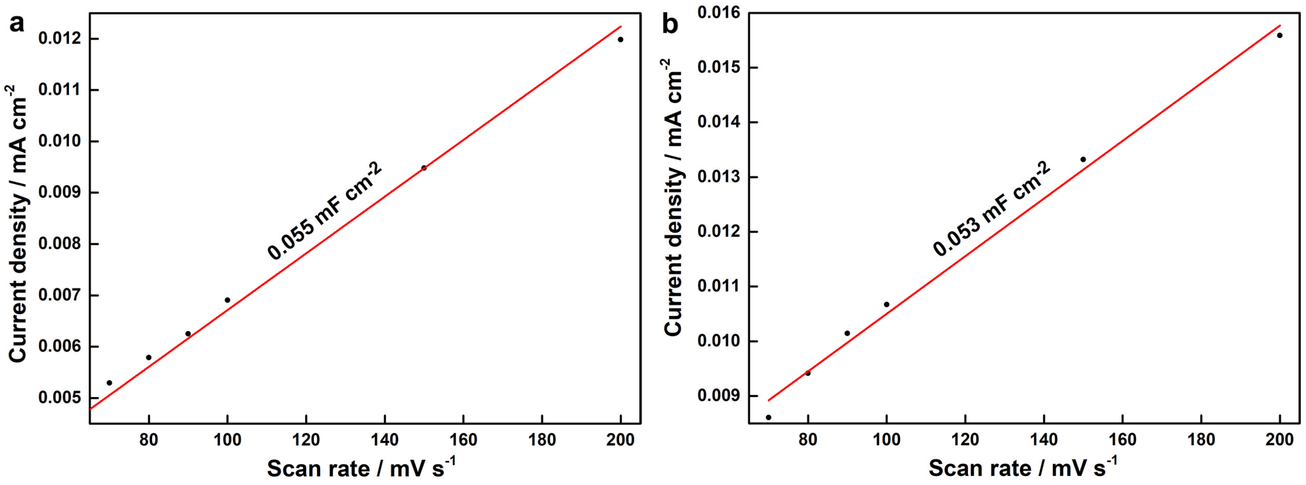

Figure S10. ECSA measurements of the NiFeB (a) and NiFe hydroxide nanosheets (b) deposited on the RDEs without supporting carbon. This figure shows the linear relationship between the current density at 0.968 V vs. RHE and the scan rate. The slopes represent the specific double layer capacitance C_{dl}, which can be converted into ECSA by ECSA = $C_{dl}/(C_s\, m)$, where C_s is the specific capacitance with the value of 0.04 mF cm^{-2} in 1 M KOH for common electrocatalysts, and m is the mass of the catalysts per geometric area of the electrode (0.1 mg cm^{-2}). The ECSAs of the NiFeB and NiFe hydroxide nanosheets can thus be calculated to be 1.375 and 1.325 m^2 g^{-1}, respectively. It is worth noting that in the absence of supporting carbon, the charge transfer between the nanosheets and the RDE becomes limited and occurs only at their direct contacting sites. As a result, the ECSA measured by this method is much lower than the physical surface area of the nanosheets. With the support-free nanosheets, the electrochemical OER occurs at their contacting sites on the electrode, not on their whole surface. Therefore, the currents in the OER can be normalized to the ECSA obtained here to reflect the intrinsic activity of the nanosheets (Figure S9).

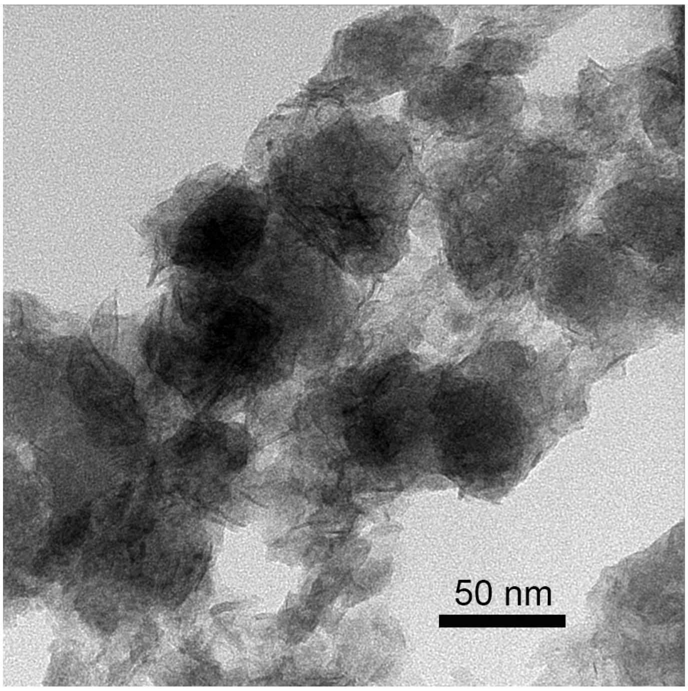

Figure S11. Typical TEM image of the NiFeB hydroxide catalyst after the chronopotentiometric test at 500 mA cm^{-2} for 130 h. The sample was obtained by sonicating the supporting carbon cloth in water. Sheet-like structures can still be observed, confirming that the morphology of the catalyst was largely retained during the harsh stability test.

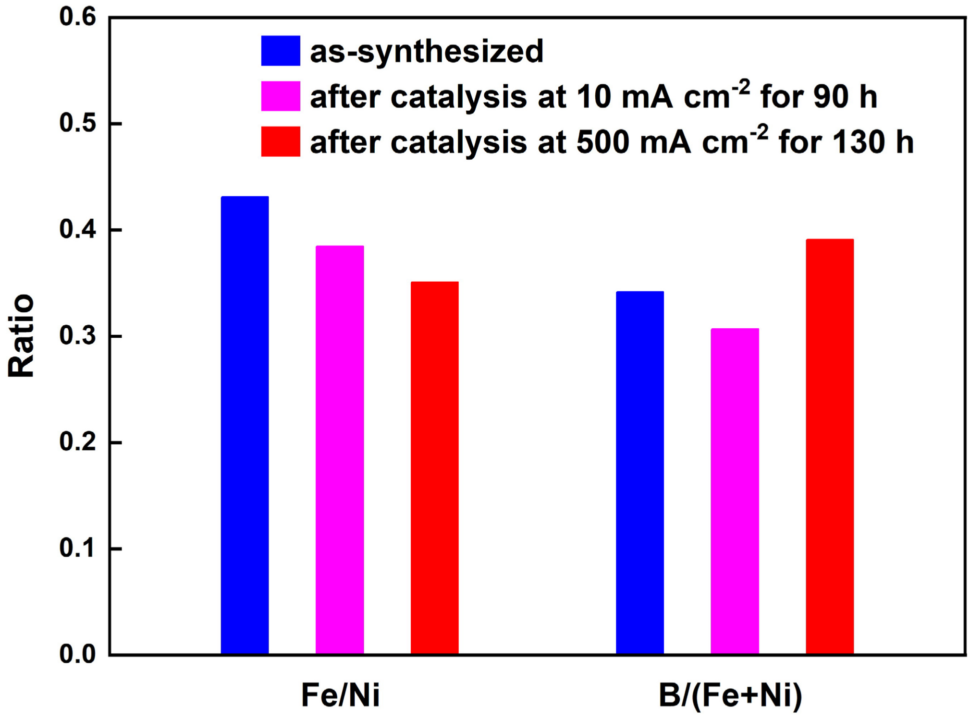

Figure S12. ICP-MS results of the NiFeB hydroxide nanosheets before and after the chronopotentiometric test at 10 mA cm^{-2} for 90 h and at 500 mA cm^{-2} for 130 h. The results show that the elements of Ni, Fe, B have been largely retained during the stability test. Data are also listed in Table S4.

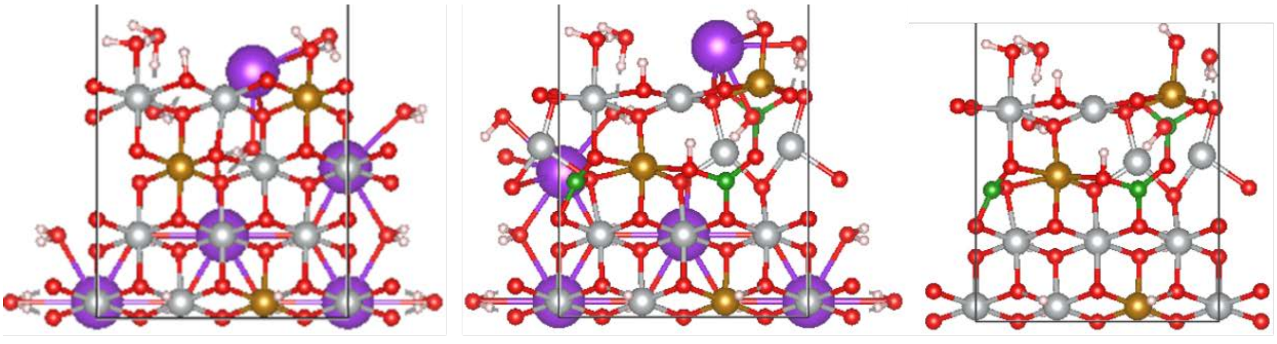

Figure S13. Models of the NiFe and NiFeB hydroxide catalysts. (a) NiFe hydroxide; (b) NiFeB hydroxide; (c) NiFeB hydroxide without adsorbing K^+. The silver, yellow, red, white, green, and purple atoms represent Ni, Fe, O, H, B, and K, respectively.

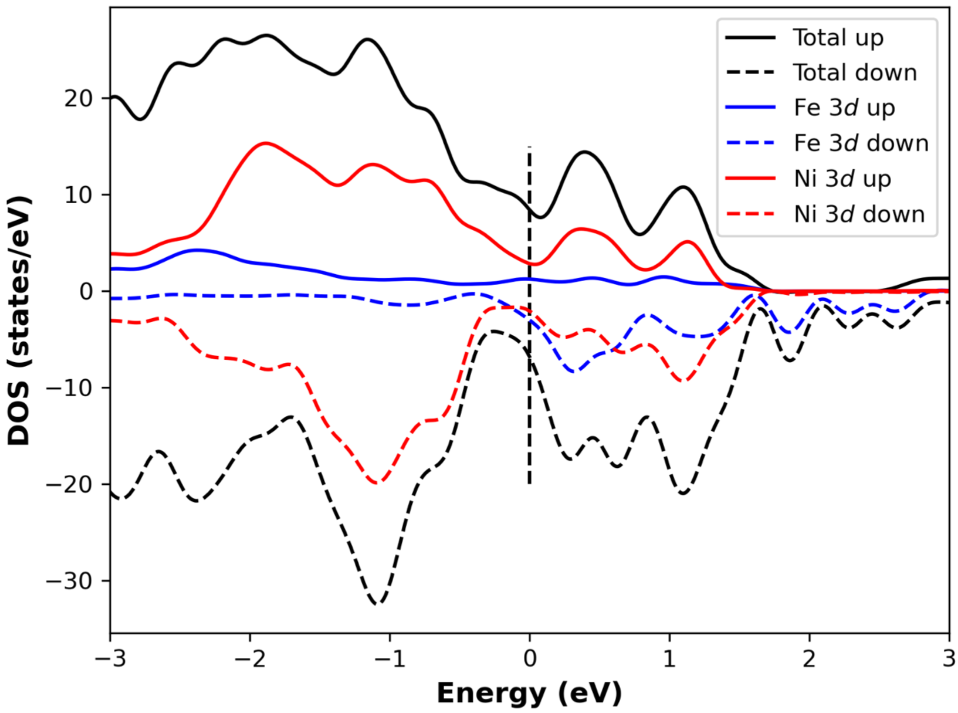

Figure S14. DFT calculated total density of state (DOS) of the NiFeB hydroxide catalyst (black) and projected DOS (pDOS) of 3*d* contributions of Ni (red) and Fe (blue). The electronic states near the Fermi level were predominantly from the Ni and Fe 3*d* orbitals, suggesting that both Ni and Fe serve as catalytic active sites for catalysis.

Table S1. ICP-MS results of the NiFe hydroxide nanosheets, NiFeB alloy nanoparticles, NiFeB hydroxide nanosheets, and NiFeB hydroxide nanosheets after ligand exchange with NO_3^-.

Samples	Fe/Ni	B/(Ni+Fe)
NiFe hydroxide	0.4431	-
NiFeB alloy nanoparticles	0.4120	0.4813
NiFeB hydroxide nanosheets	0.4311	0.3419
NiFeB hydroxide after ligand exchange with NO_3^-	0.4571	0.3711

Loading of NiFeB hydroxide nanosheets on glassy carbon electrode: 0.94 mg cm_{geo}^{-2}, containing 0.0840 mg of Ni, 0.0345 mg of Fe, and 0.0076 mg of B.

Loading of NiFe hydroxide nanosheets on glassy carbon electrode: 1.25 mg cm_{geo}^{-2}, containing 0.0828 mg of Ni and 0.0349 mg of Fe.

Table S2. Comparison of OER activity of the NiFeB hydroxide nanosheets in 1 M KOH with those of the typical catalysts reported in the literature.

Catalysts	Overpotential (mV)	Current density (mA cm_{geo}^{-2})	Reference
NiFeB hydroxide nanosheets	252	100	This work
$FeOOH_{2nm}$/LDH	174<br>270*	10<br>100	1
Exfoliated NiFe LDH	254	10	2
Fe-Co-2.3Ni-B	274	10	3
Fe-Ni-P-B-O	236<br>340*	10<br>50	4
Ni_xB/f-MWCNT	286	10	5
Hollow NiFe LDH	280	10	6
NiFeLDH-NS@defective graphene	210<br>305	10<br>50	7
NiFe-LDH hollow microspheres	239<br>320*	10<br>100	8
CoB	280	10	9
NiFe LDHs–Ni vacancy	229<br>305*	10<br>50	10
NiFe LDH-PANI	220<br>270	10<br>100	11
Amorphized LiNiFe borophosphate	215<br>280*	10<br>100	12

*Data estimated from the polarization curves of the OER.

Table S3. EIS simulation of the NiFeB and NiFe hydroxide nanosheets in electrocatalytic OER.

Model:

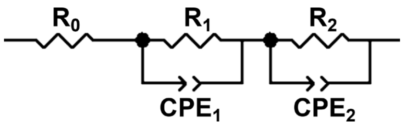

	R_0/Ω	CPE_1/mF	R_1/Ω	CPE_2/mF	R_2/Ω
NiFeB hydroxide	5.0	65.1	1.8	45.7	6.2
NiFe hydroxide	4.8	15.4	2.8	2.5	21.0

Note: The Nyquist plots were fitted by a two-*R*-CPE circuit as shown by the model above the table. Each *R*-CPE unit consists of a charge transfer resistance (R_1 or R_2, $R_2 > R_1$) and a constant phase element (CPE1 or CPE2) in parallel, where R_2-CPE_2 represents the charge transfer process in the electrocatalytic OER reaction. CPE resembles a capacitor (*C*) but the ***E–I*** phase angle is not strictly 90°. We used CPE, rather than a perfect capacitor, because it better fits the Nyquist plots in practical measurements.

Table S4. ICP-MS of the NiFeB hydroxide nanosheets before and after the chronopotentiometric tests.

Samples	Fe/Ni	B/(Ni+Fe)
NiFeB hydroxide nanosheets (as synthesized)	0.4311	0.3419
NiFeB hydroxide nanosheets after chronopotentiometric test at 10 mA cm^{-2} for 90 h	0.3847	0.3067
NiFeB hydroxide nanosheets after chronopotentiometric test at 500 mA cm^{-2} for 130 h	0.351	0.391

Supplementary References

1 Chen, J. *et al.* Interfacial interaction between FeOOH and Ni–Fe LDH to modulate the local electronic structure for enhanced OER electrocatalysis. *ACS Catal.* **8**, 11342-11351 (2018).

2 Zhao, Y. *et al.* Sub-3 nm ultrafine monolayer layered double hydroxide nanosheets for electrochemical water oxidation. *Adv. Energy Mater.* **8**, 1703585 (2018).

3 Nsanzimana, J. M. V. *et al.* An efficient and earth-abundant oxygen-evolving electrocatalyst based on amorphous metal borides. *Adv. Energy Mater.* **8**, 1701475 (2018).

4 Ren, H. *et al.* Amorphous Fe-Ni-P-B-O nanocages as efficient electrocatalysts for oxygen evolution reaction. *ACS Nano* **13**, 12969-12979 (2019).

5 Chen, X. *et al.* Ultrathin nickel boride nanosheets anchored on functionalized carbon nanotubes as bifunctional electrocatalysts for overall water splitting. *J. Mater. Chem. A* **7**, 764-774 (2019).

6 Yu, L., Yang, J. F., Guan, B. Y., Lu, Y. & Lou, X. W. D. Hierarchical hollow nanoprisms based on ultrathin Ni-Fe layered double hydroxide nanosheets with enhanced electrocatalytic activity towards oxygen evolution. *Angew. Chem. Int. Ed.* **57**, 172-176 (2018).

7 Jia, Y. *et al.* A heterostructure coupling of exfoliated Ni-Fe hydroxide nanosheet and defective graphene as a bifunctional electrocatalyst for overall water splitting. *Adv. Mater.* **29**, 1700017 (2017).

8 Zhang, C. *et al.* Hierarchical nife layered double hydroxide hollow microspheres with highly-efficient behavior toward oxygen evolution reaction. *ACS Appl. Mater. Interfaces* **8**, 33697-33703 (2016).

9 Gupta, S. *et al.* Cobalt-boride nanostructured thin films with high performance and stability for alkaline water oxidation. *ACS Sustainable Chem. Eng.* **7**, 16651-16658 (2019).

10 Wang, Y., Qiao, M., Li, Y. & Wang, S. Tuning surface electronic configuration of NiFe LDHs nanosheets by introducing cation vacancies (Fe or Ni) as highly efficient electrocatalysts for oxygen evolution reaction. *Small* **14**, e1800136 (2018).

11 Zhang, J., Zhang, H. & Huang, Y. Electron-rich nife layered double hydroxides via interface engineering for boosting electrocatalytic oxygen evolution. *Appl. Catal. B., Environ.* **297**, 120453 (2021).

12 Kwon, J. *et al.* Amorphous nickel–iron borophosphate for a robust and efficient oxygen evolution reaction. *Adv. Energy Mater.* **11**, 2100624 (2021).
